# The mystery of extreme non-coding conservation

**DOI:** 10.1098/rstb.2013.0021

**Published:** 2013-12-19

**Authors:** Nathan Harmston, Anja Barešić, Boris Lenhard

**Affiliations:** 1 Institute of Clinical Sciences, Faculty of Medicine, Imperial College London and MRC Clinical Sciences Centre, Hammersmith Hospital Campus, Du Cane Road, London W12 0NN, UK; 2 Department of Informatics, University of Bergen, Thormøhlensgate 55, 5008 Bergen, Norway

**Keywords:** vertebrate *cis*-regulation, genome evolution, conserved non-coding elements, *cis*-regulatory, evolution

## Abstract

Regions of several dozen to several hundred base pairs of extreme conservation have been found in non-coding regions in all metazoan genomes. The distribution of these elements within and across genomes has suggested that many have roles as transcriptional regulatory elements in multi-cellular organization, differentiation and development. Currently, there is no known mechanism or function that would account for this level of conservation at the observed evolutionary distances. Previous studies have found that, while these regions are under strong purifying selection, and not mutational coldspots, deletion of entire regions in mice does not necessarily lead to identifiable changes in phenotype during development. These opposing findings lead to several questions regarding their functional importance and why they are under strong selection in the first place. In this perspective, we discuss the methods and techniques used in identifying and dissecting these regions, their observed patterns of conservation, and review the current hypotheses on their functional significance.

## 1. Introduction

It has been estimated that between 5% and 10% of the human genome is evolving at rates slower than neutral [1,2]. Only 1.2% of the genome encodes proteins, and the remainder is presumed to be non-coding regions of regulatory and/or structural relevance. While there has been evidence that functionally equivalent non-coding regions can have negligible sequence similarities, and even lineage-specific transcription factor (TF) binding patterns [3,4], sequence-level conservation is still a generally applicable criterion indicative of functional conservation.

This review focuses on non-coding genomic sequences showing exceptionally high levels of similarity across species, often greater than among the exons of genes encoding perfectly conserved polypeptides [3–5]. These elements were discovered genome-wide independently by several groups in 2003–2005 [5,6] and were reported under different names and with varying conservation criteria. In the first published genome-wide report [6], the authors reported 481 sequences completely identical between human and mouse spanning 200 bp or more, whereas Sandelin *et al.* [5] and Woolfe *et al.* [7] used lower thresholds combined with a larger evolutionary distance (mammals : fish) to show that, in addition to extreme conservation, many of these elements have been conserved across more than 400 million years of vertebrate evolution. These elements also seem to represent merely the extremes of a distribution of overall highly conserved elements [1,8].

In this review, we shall use the term conserved non-coding elements, or CNEs, as a general term for all these elements. Many other names have been used by different groups, along with different conservation criteria. The conservation criteria consist of (i) a minimal sequence identity (seq. id.) between species under consideration, (ii) this identity score achieved over a minimal sequence length. Bejerano *et al*. [6] referred to elements as *ultraconserved elements* (UCEs), which are 100% conserved over their entire length, also known as *ultraconserved non-coding elements* (UCNEs) [9]. Relaxation of these thresholds enables the identification of elements over larger evolutionary distances, which are still more conserved than would be expected if these elements were neutrally evolving. Other names for these elements include *conserved non-coding elements* (CNEs), *conserved non-coding sequence* (CNS) [10], *highly conserved non-coding elements* (HCNEs) [11], *ultraconserved regions* (95% identity over at least 50 bp) [5], *extremely conserved elements* [12], *highly conserved non-coding regions* (HCNRs) [13], *hyperconserved elements* [14], *long conserved non-coding sequences* (95% over at least 500 bp) [15] and *conserved non-genic sequences* (CNS) [16]. In spite of the different names, they yield highly overlapping sets of elements representative of the same underlying phenomenon of extreme conservation. Several large-scale, publicly available sets of CNEs have been produced; they are listed in table 1.

**Table 1.** Publicly available CNE datasets.

name	CNE definition	species	dataset size	source
ANCORA [17]	70–100% seq. id. over30 or 50 bp window	Metazoa	494 human-mouse^a^	http://ancora.genereg.net/
cneViewer [18]	user-specified	human–zebrafish	73187^b^	http://bioinformatics.bc.edu/chuanglab/cneViewer/
CONDOR [19]	65% seq. id. over 40 bp	mammalian–fugu	>7000^c^	http://condor.nimr.mrc.ac.uk/
TFCONES^d^	70% seq. id. over 100 bp	human–mouse	58 954	http://tfcones.fugu-sg.org/
	65% seq. id. over 50 bp	human–fugu	2843	
UCNEbase [20]	>95% seq. id. over 200 bp (human–chicken)	18 vertebrate species	4351	http://ccg.vital-it.ch/UCNEbase
VISTA Enhancer Browser [21]	100% seq. id. over >200 bp	human–mouse	1951^c^	http://enhancer.lbl.gov

^a^100 seq. id. over 200 bp.

^b^For the minimum threshold of 50% seq. id. over 50 bp.

^c^Includes *in vivo* functional assay information.

^d^Exclusively surrounding TF genes.

The level of conservation of these sequences [6], their location within vertebrate genomes [5] and their distribution throughout the vertebrate lineage [7] suggested that these were candidates for regulatory elements important in the early stages of vertebrate development, differentiation and coordination between cells. These functions have since been experimentally confirmed for a number of elements [3,22,23].

Although these elements have been primarily identified in vertebrates, equivalent elements have been found to be pervasive throughout Metazoa, although only a few seem to be conserved between deuterostomes and protostomes [24]. Recently, CNEs have also been shown to exist in plants ([25]; see below). This suggests that these elements and the presently unknown cause of their extreme conservation are of very ancient origin, possibly going back to the origins of eukaryotic multi-cellularity.

### (a) The unexplained nature of extreme conservation

The distribution of CNEs within the genome and their level of conservation poses one of the most interesting open questions about genomic sequences: what is the reason for such extreme conservation?

**Figure 1.**
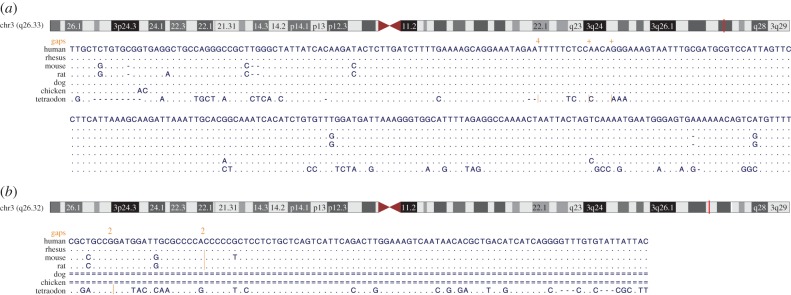
Multiple sequence alignments (Multiz alignment of 46 vertebrate genomes) of a set of sequences that are highly conserved over vertebrates. Dots represent bases that are identical to the human GRCh37/hg19 assembly and orange lines represent gaps. (*a*) Alignments for a CNE near the *SOX2* locus, chr3: 180 462 261–180 462 515 and (*b*) a CNE located at chr3: 177 077 799–177 077 901, which is missing in dog and chicken.

To date, no plausible explanation has been proposed for either the source of selective pressure or a potential direct mechanism which would result in such a high level of conservation as seen in a subset of conserved non-coding elements (see the examples in figure 1). No imaginable combination of overlapping TF binding sites (TFBSs) could account for them, and the accumulating ChIP-seq data provide no evidence for massive amounts of combinatorial TF binding to those elements. Furthermore, no known complementary RNA products exist that could target them across their entire conserved length, and no plausible mechanism of active maintenance of the sequence has been proposed. However, their pervasive nature and implication in developmental and multi-cellular processes suggest that the unknown source of conservation holds a key to understanding the regulation of development and differentiation in general.

This paper aims to review what is known about CNEs, their currently identified functional and structural features, conservation patterns and their prevalence in the tree of life. Finally, we provide an overview of current opinions on the mechanisms of their emergence, conservation and evolution.

## 2. General features of extremely conserved elements

### (a) Distribution within genomes and its consequences

**Figure 2.**
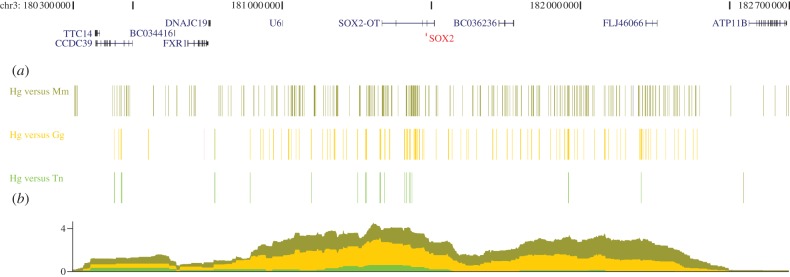
Overview of the *SOX2* locus, its associated gene desert and its local neighbourhood, specifically the 2.4 Mb region on human chr3 centred around the *SOX2* gene. (*a*) Location of CNEs flanking human *SOX2*, present between human (Hg) and mouse (Mm) (90% identity over 50 base pairs—shown in dark green), human and chicken (Gg; 90% identity over 50 base pairs—shown in yellow) and human and tetraodon (Tn; 70% identity over 50 base pairs—shown in light green). (*b*) As the distance increases from *SOX2*, the density of HCNEs decreases dramatically.

The location and distribution of CNEs within a genome is not random: they appear in clusters, more often around genes encoding crucial regulators of early development than expected by chance [5–7,26]. The distribution and density of CNEs within the vicinity of the developmental gene *SOX2* is shown in figure 2. Even though there is virtually no sequence homology between the CNEs identified among the genomes of the *Drosophila* genus and those identified in vertebrates, they tend to be associated with the same functional classes of genes. These elements are also enriched close to genes involved in ion flow across membranes and cell–cell communication, but are underrepresented near housekeeping genes [27]. CNEs are also enriched in 3′ untranslated regions (3′-UTRs) of regulatory genes (less so in invertebrates) [1].

There is ample evidence that CNEs are required to be kept in *cis* with the gene they are involved in regulating (their *target gene*). This has constrained how the genome is organized [28,29] and has led to the maintenance of large regions of synteny conserved over large evolutionary distances, populated by a set of CNEs targeting one particular gene, referred to as *genomic regulatory blocks* (GRBs) [30–32]. The neighbourhoods of many of these target genes are devoid of other genes (gene deserts) and are heavily populated by CNEs [22] (figure 2), although there exist many examples where they contain *bystander* genes which contain CNEs of target genes but appear not to be responsive to regulation by them, reflecting differences in their promoter architecture [33] or the importance of the structural organization of the locus [34]. The distribution of CNEs within a GRB tends to a show a high density of CNEs around the (predicted or experimentally demonstrated) target gene (including its introns and the introns of bystander genes), with the density decreasing at larger distances from the promoter of the target gene. In total, CNE clusters can span up to a couple of megabases around their target genes [5,35].

Considering these elements have been linked to key developmental regulators, it has been proposed they might be used as indicators of loci of yet undiscovered or unannotated developmental genes [5,17]. A subset of developmentally controlled microRNAs were also found to be associated with clusters of deeply conserved CNEs [36], again linking these elements with a particular functional subset of genes.

Several studies identified regions that seem to be mutually exclusive with clusters of CNEs. CNEs were depleted in regions with segmental duplications and copy number variations [37]. In addition, many but not all mammalian loci containing clusters of CNEs were shown to be depleted for transposons [38]. The loci that were depleted in retrotransposon insertions were associated with developmental TFs, suggesting that the *cis*-regulatory architecture of these genes is unable to tolerate insertions of this type.

### (b) Prevalence of extreme conservation across species

The human elements most highly conserved in other species are common to all vertebrates, making vertebrate model organisms, especially mouse, zebrafish and medaka, convenient model organisms for *in vivo* functional assays of CNE effects on target genes. At larger evolutionary distances, the number of conserved non-coding elements rapidly declines—e.g. between human and sea lamprey only 76 CNEs were reported [39], and only 56 between human and the early branching chordate *Branchiostoma floridae* (amphioxus/lancelet) [40]. Thus, it is not surprising that there is virtually no non-coding sequence similarity to invertebrate CNEs for the orthologous genes, including urochordates as the closest relatives [27].

Recently, very rare individual CNEs were found to show conservation (at lowered thresholds) across larger evolutionary distances. A small number of CNEs were found near the *Hox* locus in amphioxus versus chicken/mouse [41] or amphioxus versus mouse/*Ciona* [42] comparisons. Clarke *et al*. [24] identified two regulatory elements conserved between deuterostomes and protostomes which were found to remain in synteny with their respective genes. In addition, several CNEs have also been identified to show marginal similarity between *D. melanogaster* and humans [43].

While most CNE studies focused on comparing humans with mammalian or other vertebrate species, several studies found equivalent sets of elements conserved across invertebrate genomes when comparing genomes across a suitable range of evolutionary distances. Equivalent elements were found to be highly conserved across worms of the *Caenorhabditis* genus [27]*, Drosophila* genus [31,44], across different mosquito genomes [31] and between two species of *Ciona* (B. Lenhard 2013, unpublished observation)*.* Despite the lack of sequence-level similarity across the different clades, these clade-specific sets of elements have many features in common; they occur in genomic clusters around genes whose protein products themselves regulate embryonic development and differentiation, they impose the constraints of genome rearrangements within those clusters, and the loci of their target genes are characterized by broad Polycomb repression and associated broad H3K27me3 marks when they are being held in an inactive state [31].

Siepel *et al.* [1] analysed conserved elements (in both coding and non-coding regions) by aligning them within clades: five vertebrates, four insect species, seven *Saccharomyces* and two *Caenorhabditis* species. Comparing all conserved elements showed an increase in total element frequency among smaller, more compact genomes and larger fractions of non-coding elements in organisms with more complex genomes, i.e. vertebrates.

Finally, elements with similar properties have been reported in plant genomes [25,45]. Many were found in the vicinity of TF genes that regulate plant development, including those that do not have orthologues in Metazoa. This strengthens the hypothesis that clusters of these elements are a functional feature of the regulation of genes involved in development and differentiation, and suggests an even more ancient origin for them.

### (c) General sequence properties of conserved non-coding elements

Despite extensive early efforts to find them, there is little evidence for the existence of sequence-level features common to CNEs as a class of genomic elements. CNEs show a biased AT (adenine and thymine) content with (i) increased total AT content within CNEs when compared with surrounding sequences, (ii) a sharp increase in AT frequency at CNE boundaries and (iii) a sharp decline in AT frequency on the boundaries of sequences flanking CNEs [46]. The strength of this pattern depends on the background properties of the genome sequence in question; it is particularly strong in CNEs of genomes with relatively high GC content—fugu, *Caenorhabditis elegans* and *Drosophila melanogaster*—and less prominent in mammals [27]. Finally, the AT content of CNEs differs significantly from average gene surroundings, suggesting selective pressure for this sequence feature [46].

While most studies identify CNEs as the largest stretches of non-coding sequences to satisfy a defined sequence identity threshold, Hare *et al.* [47] attempted to identify what remains in terms of their functional content after long times of evolutionary separation. They compared six species of sepsids (insects that belong to the same order—Diptera—as *Drosophila*) with *D. melanogaster*, which diverged from them approximately 100 million years ago, ensuring the identification of highly diverged regulatory sequences which still drive highly similar expression patterns. They showed that the enhancer of the *eve* gene contains highly conserved small blocks of only 20–30 nucleotides, enriched in overlapping TFBSs. This finding is in agreement with the billboard model of *cis*-regulatory modules [48], which proposes that the exact number and order of TFBSs is not necessary for the correct enhancer effect on the target gene. These 20–30 nucleotide clusters of TFBSs may, however, be the smallest blocks selection acts upon in functional CNEs [47]. Woolfe *et al.* [7] found consistent ordering and mutual positioning of CNEs within vertebrate genomes, suggesting their (yet undetermined) structural/organizational role, although the 56 CNEs conserved between amphioxus and vertebrates show some evidence of shuffling with respect to order and orientation [40].

These studies show that CNEs can have important regulatory functions, although we still cannot account for the pattern or extent of conservation at closer distances. It seems that, as of yet, there is no consensus on the minimal set of features defining an enhancer with a conserved output to be selected against. However, these analyses, along with the relatively high abundance of CNEs in gene deserts, suggest structural importance, making it necessary to view these elements as more complex than just a collection/ordered combination of TFBSs.

### (d) Biological function of conserved non-coding elements

The ability of a CNE to drive expression of a gene is typically tested *in vivo* using transgenic assays, most commonly in mouse [3] or zebrafish [4,49]. A majority of the tested CNEs act as enhancers in reporter constructs [3]. The probability that a conserved sequence has enhancer activity is related to its level of conservation [3] and the density of other conserved sequences in the surrounding locus [50]. Transgenic assays of a number of CNEs lacking enhancer activity revealed that they were able to function as enhancer-blocking insulators [51]. A handful have also been found to be involved in regulating other transcription-related processes, such as splicing [52] and RNA editing [53].

In addition to the sequence of these elements being highly conserved, the majority of CNEs that act as enhancers also show functional conservation over the entire clade in which they are conserved, and very often beyond. A set of lamprey and human CNEs located near the *EBF3* gene has been found to upregulate GFP expression in the same set of neurons in zebrafish [39]. However, the expression patterns they are driving in different species can vary dramatically [54]. Transfection of CNEs identified across multiple phyletic groups has found that these elements can still drive expression, although at slightly different development stages [55].

While CNEs detected in three clades independently (insects, worms and vertebrates) do not share sequence similarity, they often associate with and regulate the same set of (often crucial developmental) genes in all three groups [17,27]. This suggests that the involvement of highly conserved non-coding elements in the precise regulation of these genes is crucial for the body plan development within a phylum, whereas recycling regulatory states using the same pool of enhancer sequences in different contexts might be the driving force in the emergence of different body plans during evolution [56]—a phenomenon termed regulatory interaction re-wiring by Vavouri *et al.* [57]. Tunicates display a typical chordate body plan using a highly diverged set of conserved elements when compared with other chordates [58]; however, the elements still cluster around the same types of genes as in other chordates and indeed other Metazoa.

Given their extreme levels of conservation over long stretches of genomic sequence, it is expected that these elements play important and irreplaceable functions in early development. Surprisingly, at least in some cases, the deletion of large clusters of CNEs yields viable mice with no obvious deleterious phenotypic changes, as shown by transgenic mouse assays [59]. There have been several recent indications that some of the CNEs are phenotypically redundant, or only have phenotypes that are detectable over many generations [60–62]. To sum up, it is impossible to infer functional conservation from sequence conservation and vice versa [63–65].

More than one-third of top disease-associated regions coming out of genome-wide association studies do not contain any coding sequences [66], thus indicating a common role of non-coding sequences in disease [67,68]. Many of those regions are spanned by multiple CNEs [69], making it possible that a number of genetic diseases are associated with CNE function. In order to shed more light on the role of CNEs within the genome, it is thus crucial to look into the evolutionary background of these elements.

## 3. Origins and evolutionary dynamics of conserved non-coding elements

### (a) Purifying selection versus mutational coldspots

One of the first explanations proposed for the existence of CNEs is that they are located within regions associated with very low rates of mutation (mutational coldspots). However, these elements exhibit features which suggest that they are constrained by extreme levels of purifying selection—a lower than expected single nucleotide polymorphism density [6,70], and a derived allele frequency significantly shifted towards ancestral alleles [71]. The frequency of germline mutations in a set of vertebrate CNEs has been found to be similar to that of other genomic regions, suggesting that mutations in these regions can occur, but are actively selected against [15]. Similar signatures of purifying selection have also been identified in insects [72], suggesting that the same constraints apply to these elements across Metazoa. However, although the majority of evidence is in support of these elements being under selection, the observations that the knockdown of some of these sequences leads to viable mice [59] and that a number of CNEs accumulate fewer mutations than their flanking regions in colorectal cancer [52] have raised as of yet unanswered questions regarding their functional importance and the source of their observed levels of selection.

### (b) Emergence and recruitment of conserved non-coding elements

The CNEs in a genome are generally unrelated on the sequence level—the exception being CNEs whose common ancestor can be traced back to a whole-genome duplication (WGD) [7,73–75]. This reflects that CNEs appear to have been derived from a multitude of different sources, including former exons [75,76], introns [44], mobile elements [8,77] and ancient repeats [78].

Some CNEs have been found to originate from retrotransposons [8] and other classes of mobile elements [77], which have been exapted and have since come under selection (reviewed in [79]). This finding is in contrast to the findings of Simons *et al.* [38], where regions of the genome containing developmental regulatory genes were found to be depleted in transposon insertions. However, it appears that exaptation of these elements can be identified only for ancient insertions, indicating that selection against recent insertions is occurring and is potentially responsible for their depletion around specific genes. It may be that some retrotransposon insertions are preferentially retained in certain contexts as they are useful in creating new *cis*-regulatory elements. Certain families of transposable elements appear to have sequences that are easily mutated into TFBSs [80]; however, it has been shown that transposable elements from all superfamilies have the ability to come under extreme levels of selection [81]. Hundreds of sequences from the MER21 family of ancestral repeats have been found to have been exapted during evolution [78] and are now identifiable as CNEs within the human genome. These sequences appear to contain a set of even more highly conserved short subsequences, which correspond to putative and known binding motifs, although the authors provided no experimental evidence of TF binding.

A highly conserved exonic enhancer involved in hindbrain development has been found to lie within a conserved element found in all vertebrates [76]; the element itself extends into the flanking introns. This implies that the same selective pressure that can be applied to non-coding elements can also be present within coding regions and overlap with the selective pressure acting to conserve the underlying protein sequence.

In conclusion, although certain types of sequence have a higher propensity to gain regulatory functions, there is no evidence that any specific type of sequence element has an increased probability of being recruited as a CNE. It appears that any sequence within the response range of a gene responsive to long-range regulation, once it provides some important regulatory function, has the potential to become recruited as a CNE.

There is evidence that some CNEs have been recruited either through a process of gradual accumulation or in discrete waves. However, the (still) limited sampling of the vertebrate phylogenetic tree makes it difficult to distinguish between these models. Analysis of the vertebrate phylogeny has found that CNEs appear to be recruited in a lineage-specific manner—with approximately 40% of extant eutherian CNEs being present before the divergence of ray-finned fishes from cartilaginous fishes, 12% appearing in the bony vertebrates, 18% in the tetrapods, and 16% and 10% appearing in the amniotic and therian ancestor, respectively [82]. It appears that CNEs evolved rapidly in the early vertebrate lineage [73], and since the divergence of tetrapods and the teleosts, many tetrapod CNEs have been mutating at an extremely low rate [83]. By analysing substitution rates observed in CNEs, Kim *et al.* [84] found that two-thirds of CNEs evolved at a rate consistent with a one-parameter model; however, the remainder showed branch-specific changes in the observed mutation rate. This suggests that the adaptive evolution of CNEs may occur in short bursts, and that the selective constraints imposed on certain sets of CNEs has not remained constant during mammalian evolution.

Ryu *et al.* [43] identified CNEs from several phyla and investigated their patterns of evolution. CNEs were identified not only between higher eukaryotes, but also between species in more primitive phyla (e.g. Porifera and Cnidaria). In all of the examined phyla, CNEs were found to be recruited in clusters around genes belonging to equivalent functional groups. These elements could be clustered into their respective lineages based on their sequence similarity, with no identifiable sequence conservation across distant lineages. Ryu *et al.* suggested that each group of CNEs arose independently in the ancestors of different phyla, and following divergence of that lineage, came under selection and became fixed. However, any mechanism of selection shared across different phyla should have been in place already in their last common ancestor—including the source of purifying selection—so it is likely that the species that lived many hundreds of millions years ago already possessed their own set of CNEs, which diverged by slow but eventually complete turnover in different lineages after their separation. For a further discussion of CNE turnover, see below.

### (c) Patterns of loss, gain and divergence of conserved elements

Lowe *et al.* [85] proposed that, within vertebrates, there have been three distinct periods of CNE recruitment around specific groups of genes. They suggest that this pattern is the result of regulatory innovations, which led to important phenotypic changes during vertebrate evolution. Prior to the divergence of mammals from reptiles and birds, it appears that CNEs were preferentially recruited near TFs and their developmental targets. This was followed by a gradual decline in recruitment near these genes, accompanied by an increase near proteins involved in extracellular signalling, and then an increase in placental mammals near genes responsible for post-translational modification and intracellular signalling. An analysis of CNE gain in the primate and rodent lineage has found that CNEs are either recruited near genes which have not previously been associated with CNEs, or are added near genes which are already flanked by CNEs [86]. The interpretation was that the first set of genes is enriched in functions pertaining to nervous system development, whereas the latter contains genes involved in transcriptional regulation and anatomical development. A set of genes involved in DNA binding and transcriptional regulation was found not to gain new elements in addition to pre-existing ones.

During evolution, the flanking sequences of a CNE can show substantial levels of divergence, whereas a core region remains highly conserved. Comparisons of a well-conserved element identified in *Tetraodon* show that this element is flanked by lineage-specific mutations in the mammalian and fish lineages. The degree of sequence divergence in the regions surrounding a core CNE [87,88] has led to these elements being proposed as markers for phylogenetic studies, successfully resolving the phylogeny of non-model organisms, in addition to reconstructing the primate and placental tree. Comparisons of human, mouse and primate CNEs suggest the phenomenon of ultraconservation is fragile [89], and that once a mutation within a CNE has become fixed, it appears that the element becomes more susceptible to gaining additional mutations.

Despite being under such high levels of selection, CNEs do show patterns of lineage-specific loss. In several cases, loss of a CNE was shown to be accompanied by detectable alterations in an organism's phenotype and fitness [90,91], further reinforcing their functional importance. It is therefore expected that CNE loss, which negatively affects the fitness of an organism, will be selected against and will not become fixed in populations. Within the rodent lineage, mammalian-specific CNE loss has been estimated to be 300 times less probable than the loss of neutrally evolving sequence [92]. An examination of CNE loss in mammals [93] found that independent CNE loss occurs non-uniformly across the mammalian lineage, with CNEs that are shorter, younger and under less constraint showing a higher likelihood of being lost. The rate of conservation of CNEs dating back to the amniote ancestor is different between mammals and reptiles [94], which have lost similar numbers of CNEs but at different rates.

The current understanding of *cis*-regulatory evolution proposes that loss of a regulatory element can only occur once the selective pressure on that element is either absent or sufficiently relaxed. This situation can occur by (i) the creation of a new element, which performs the same function, making the original element redundant (known as turnover), (ii) the loss of the pressure on the tissue/phenotype that the enhancer is responsible for or (iii) the loss of the gene it regulates. CNEs are absent from chrY [7], with the exception of the *SHOX* locus in its pseudo-autosomal region. *SHOX*-associated CNEs are well conserved between human, dog and fish. Owing to the loss of the *SHOX* gene in the mouse lineage, no CNEs are identifiable between the human and mouse chrY [15]. However, the loss of the CNE-associated gene is extremely rare and can explain only a small fraction of the observed losses [93].

### (d) Turnover of *cis*-regulatory elements and conserved non-coding elements

The conservation of the expression pattern of a gene is not dependent on the sequence conservation of its regulatory elements [47,63]. It has been found that the *cis*-regulatory architecture of the *yellow* gene in *Drosophila* has changed multiple times during evolution [95]: both the sequence and position of the various enhancers have changed. In addition, enhancers that were responsible for driving expression in specific tissues had changed their genomic location. This region shows no evidence of segmental duplications and transpositions, suggesting that the observed patterns of turnover probably occur owing to the gradual accumulation of mutations, which result in the *de novo* gain and loss of TFBSs. Small sequence changes can inactivate existing *cis*-regulatory elements, and can generate new *cis*-regulatory elements from non-regulatory sequences [96].

Mammals may contain similar amounts of functional sequence, despite loss of many conserved sequences [2,97], suggesting that turnover of functional non-coding sequences is both prevalent and occurring at different rates. The lack of non-coding sequence conservation between different phyla, together with differences in retention of these elements across lineages and between closely related species, suggests that CNEs have been subject to turnover since their initial recruitment.

**Figure 3.**
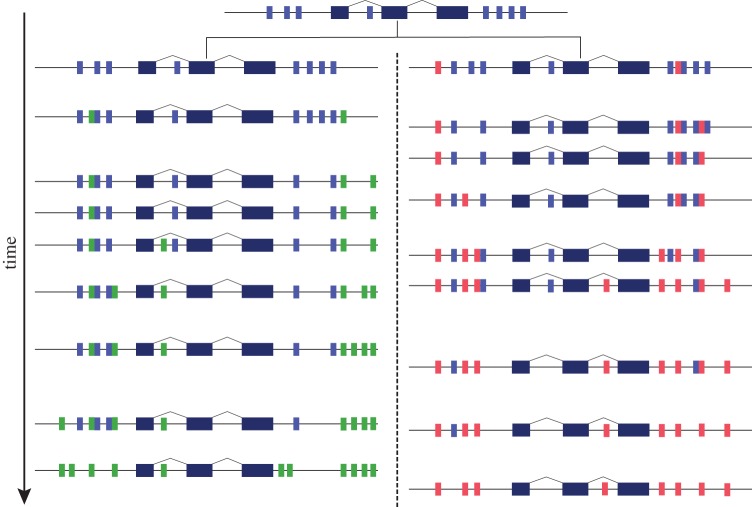
Schema of our proposed model of CNE turnover. In the common ancestor of two lineages, *cis*-regulatory elements (shown in light blue) were recruited within the proximity of a gene which was required to be under a specific form of regulation. Over time, other elements were sequentially recruited in both lineages (shown in green and red) and the corresponding ancestral elements were lost. This process continued until all of the elements in the extant set of CNEs no longer contain any of the set of ancestral elements, and these elements are no longer recognizable between lineages. This results in CNEs changing in position and arrangement within the locus, as well as gaining lineage-specific elements.

We propose that all extant CNEs are not indispensable, but that given an adequate amount of time, all of these elements will eventually be replaced by new ones, which provide equivalent functions (figure 3). On the whole, CNEs in a genome are unrelated at the sequence level [7], and they are absent from regions of segmental duplications and copy number variation [37]. This suggests that duplications involving them are strongly selected against in the cases where the duplicate elements still affect a target gene, or are lost rapidly where they do not. On the other hand, their occurrence in the introns of neighbouring genes and recruitment from diverse existing genomic elements suggests that they appear by a gradual process of mutation, recruitment and selection. The different rates at which these elements turn over reflects differences in the levels of selective constraint, and how likely it is that a replacement element can be recruited without interfering with the function of existing ones.

It has been proposed that CNEs reflect the parallel evolution of regulatory elements for important developmental regulatory genes in different groups [27]. The following model of CNE recruitment and turnover can directly explain this proposition. At some stage during evolution, ancestral developmental *cis*-regulatory elements appear to have been recruited from sequences near specific sets of genes. These elements provided regulatory innovations that were necessary for development of multi-cellular organisms. Within each of these regions, there was the potential for sequences to gain important functions and for selection on existing elements to be relaxed, allowing them to diverge and be turned over. During evolution, additional genes were recruited to developmental regulatory networks, leading to increasingly complex developmental and morphological features. The presence of clusters of CNEs near orthologous genes in species separated by large evolutionary distances argues for this hypothesis, as does the limited number of CNEs which are found between phyletic groups [24]. In the cases where there are no elements conserved between two distant species around a specific orthologous locus, while they clearly exist between each of the species and its closer relatives, the ancestral elements have completely turned over and are no longer identifiable.

Not all key regulatory elements involved in development are CNEs, which leaves the question of the link between developmental function and source of extreme selective pressure unresolved. As an example, both conserved and non-conserved regulatory sequences are required for controlling developmental genes in the germ layer of zebrafish [98]. It may be that lineage-specific CNEs have the same function as elements that have been lost even if their sequences are not homologous [86]. Some of the new lineage-specific CNEs that are generated by turnover may not be contributing to lineage-specific changes but are required for maintaining important patterns of gene expression as substitute or partially redundant elements.

## 4. Mechanism of conservation and unexplored potential roles of extremely conserved elements

Despite the amount of research into CNEs, there is as of yet no unifying model relating their functional properties with their observed evolutionary dynamics and the extent of conservation. Currently, there is no known biological or biochemical function that requires such large elements to be under such high levels of sequence constraint. Several hypotheses have been suggested to explain their presence based on their potential functions and patterns of conservation; however, serious objections can be raised to all of them.

### (a) Hypotheses on the origin of conservation

Based on experiments that support the hypothesis that these elements act as developmental stage-specific enhancers, one would hope that existing models of enhancer architecture would help illuminate this question; however, they only serve to make the issue more perplexing. Enhancers have been classified into distinct groups based on the arrangement of their constitutive TFBSs and the degree of cooperativity between bound TFs [99]. The enhanceosome [100] model features a strict pattern of TFBSs, which in some cases enables cooperativity between bound TFs. Such an arrangement could potentially span over a large number of nucleotides and be subject to high levels of selection. However, the enhanceosome model only requires sequence conservation at the level of binding sites and their interleaving distances—it does not require conservation of the inter-site sequences. As such, this would not lead to the observed long stretches of extreme conservation.

The degeneracy of TFBSs is typically thought to suggest that DNA–protein interactions are promiscuous and do not require a perfect binding site. However, mutations within *cis*-regulatory elements can have large and unexpected effects [101]. Phenotypic and morphological evolution can be directly influenced by mutations which have a small effect size [102]; however, these mutations can be selected against. It may be that mutations within these elements have effects that are subject to extreme levels of purifying selection. One potential explanation is that these elements contain overlapping TFBSs, where alteration of one nucleotide position has effects on multiple overlapping TFBSs and may affect nucleosome positioning and retention. Given the levels of TFBS degeneracy and the weak sequence requirements for nucleosome positioning signal, this would require an extremely dense overlap of functional elements that has never been observed at any regulatory element so far. On the contrary, despite the rapidly growing volume of TF binding and histone modification data [103–105], there is no evidence that CNEs that act as enhancers bind a larger number of DNA binding proteins or have different histone modification marks than regulatory elements lacking their level of conservation. Indeed, for many elements, over a large number of cellular conditions and embryonic stages tested, there is no evidence for any enhancer-associated features from the binding and histone modification data.

The size of these elements and their patterns of divergence and fragmentation suggest that these may not only have one specific function, but are multi-functional. The flanking elements may be important in determining the function and specificity of a CNE [39,106]. This may suggest that these elements are pleiotropic and under selection owing to multiple coinciding functions. However, this hypothesis still relies on TF binding and chromatin features as sources of selective pressure and as such also fails to explain the extent of conservation.

The hierarchical nature of the developmental genetic regulatory networks (GRNs) [107] has suggested that these elements may be involved in the early stages of embryonic development or during a specific period during development [56]. It has been proposed that CNEs may be responsible for regulation at the end of gastrulation (the *phylotypic stage*), where patterns of gene expression appear to be highly conserved between species [108], the recruitment and persistence of these elements being due to selective pressure to maintain the observed patterns of expression [109]. Furthermore, the enhancers used at the end of gastrulation show a significant increase in the degree of sequence conservation [110]. However, even this hypothesis still supposes that selection is acting at the level of TFBSs, and predicts that all the most conserved CNEs are involved in transcriptional regulation during the phylotypic stage, when this is clearly not the case. Another potential way for CNE evolution to be constrained by the structure of the GRN is that they could potentially be recruited to act at different levels of the GRNs, having multiple functions and potentially large pleiotropic effects [55]. This explanation suffers from the same problem as the previous ones.

In addition, it has been suggested that these elements may be involved in homologous recombination [27,111], which would provide an active mechanism for the elimination of differences between two alleles of the same element. However, this or any other active mechanism would require them to function primarily in the germline, which does not match what is known about their biological function, although, because the known biological functions cannot explain the level of conservation, this hypothesis cannot be ruled out at the present time despite the lack of any experimental evidence.

The use of chromosome conformation assays have identified that some CNEs appear to be involved in *cis-* and *trans*-interactions with other CNE-rich regions of the genome [112]. CNEs were found to interact with promoters of genes as well as other CNEs. This suggests that these interactions may be involved in the regulation of a set of functionally related genes or in the formation of higher-order chromatin structures. Dimitrieva & Bucher [9] investigated the patterns of CNE retention and loss following WGD and suggested that the majority of CNEs are retained in *cis* with one copy of the duplicated gene while having been completely lost from the other copy. While this reason for their conservation is appealing, the existence of these interactions has only been reported in one study and it remains to be seen whether these interactions are prevalent and functional. Recent results have suggested that the conformation at developmental loci is highly divergent across mammals [113], which may point to CNEs been involved in the conservation of a set of interactions and higher-order chromatin structure.

### (b) Clues about function from chromatin and epigenetic data

As noted earlier, the analysis of the recently released ENCODE data suggests that there is nothing special about CNEs that set them apart from other regulatory elements in terms of their epigenetic features. However, it has been shown repeatedly that the genes that are regulated by them (and around which they form dense clusters) are associated with special patterns of histone modifications and TF binding. Intriguingly, it has been shown in both human [114,115] and zebrafish [116] that these genes are the most prominent subset of genes that retains histones and histone modifications in the sperm genome. These genes in sperm typically have bivalent promoters (overlapping H3K4me3 and H3K27me3 marks) as well as locus-wide H3K27me3 marks that often cover the entire gene [117]. While these observations do not tell anything about the role of CNEs in sperm or spermatogenesis, they have the ability to generate hypotheses about the possible role of CNEs in the germline.

## 5. Conclusion and perspectives

Since their discovery, research into CNEs has led to several important findings regarding their functional importance and evolutionary dynamics. However, despite 10 years of research, there has been virtually no progress towards answering the question of the origin of these patterns of extreme conservation. A number of hypotheses have been proposed, but most rely on modes of DNA : protein interactions that have never been observed and seem dubious at best. As a consequence, not only do we still lack a plausible mechanism for the conservation of CNEs—we lack even plausible speculations.

It is clear that selection is acting on more than the just the sum of the constitutive TFBSs within a CNE. We expect CNEs to be found throughout all of Metazoa and even more broadly throughout multi-cellular organisms. Given the ancient origins of CNE-associated developmental regulation, the model that includes recruitment, selection over large periods of time and turnover is a more parsimonious explanation for their evolutionary dynamics than their independent occurrence in parallel lineages. Further work on the evolutionary dynamics of these elements and new hypotheses about the origin of their conservation is needed in order to begin to understand the mechanism behind this mysterious and fascinating feature of multi-cellular genomes.

## References

[1] Siepel A 2005 Evolutionarily conserved elements in vertebrate, insect, worm, and yeast genomes. Genome Res. 15, 1034–1050 (doi:10.1101/gr.3715005) 16024819 10.1101/gr.3715005 PMC1182216

[2] Meader S Ponting CP Lunter G 2010 Massive turnover of functional sequence in human and other mammalian genomes. Genome Res. 20, 1335–1343 (doi:10.1101/gr.108795.110) 20693480 10.1101/gr.108795.110 PMC2945182

[3] Pennacchio LA 2006 *In vivo* enhancer analysis of human conserved non-coding sequences. Nature 444, 499–502 (doi:10.1038/nature05295) 17086198 10.1038/nature05295

[4] Taher L McGaughey DM Maragh S Aneas I Bessling SL Miller W Nobrega MA McCallion AS Ovcharenko I 2011 Genome-wide identification of conserved regulatory function in diverged sequences. Genome Res. 21, 1139–1149 (doi:10.1101/gr.119016.110) 21628450 10.1101/gr.119016.110 PMC3129256

[5] Sandelin A Bailey P Bruce S Engström PG Klos JM Wasserman WW Ericson J Lenhard B 2004 Arrays of ultraconserved non-coding regions span the loci of key developmental genes in vertebrate genomes. BMC Genomics 5, 99 (doi:10.1186/1471-2164-5-99) 15613238 10.1186/1471-2164-5-99 PMC544600

[6] Bejerano G Pheasant M Makunin I Stephen S Kent J Mattick JS Haussler D 2004 Ultraconserved elements in the human genome. Science 304, 1321–1325 (doi:10.1126/science.1098119) 15131266 10.1126/science.1098119

[7] Woolfe A 2005 Highly conserved non-coding sequences are associated with vertebrate development. PLoS Biol. 3, e7 (doi:10.1371/journal.pbio.0030007) 15630479 10.1371/journal.pbio.0030007 PMC526512

[8] Bejerano G Lowe CB Ahituv N King B Siepel A Salama SR Rubin EM James Kent W Haussler D 2006 A distal enhancer and an ultraconserved exon are derived from a novel retroposon. Nature 441, 87–90 (doi:10.1038/nature04696) 16625209 10.1038/nature04696

[9] Dimitrieva S Bucher P 2012 Genomic context analysis reveals dense interaction network between vertebrate ultraconserved non-coding elements. Bioinformatics 28, i395–i401 (doi:10.1093/bioinformatics/bts400) 22962458 10.1093/bioinformatics/bts400 PMC3436827

[10] Boffelli D Nobrega MA Rubin EM 2004 Comparative genomics at the vertebrate extremes. Nat. Rev. Genet. 5, 456–465 (doi:10.1038/nrg1350) 15153998 10.1038/nrg1350

[11] Lindblad-Toh K 2005 Genome sequence, comparative analysis and haplotype structure of the domestic dog. Nature 438, 803–819 (doi:10.1038/nature04338) 16341006 10.1038/nature04338

[12] Mouse Genome Sequencing Consortium, Waterston RH 2002 Initial sequencing and comparative analysis of the mouse genome. Nature 420, 520–562 (doi:10.1038/nature01262) 12466850 10.1038/nature01262

[13] de la Calle-Mustienes de E 2005 A functional survey of the enhancer activity of conserved non-coding sequences from vertebrate *Iroquois* cluster gene deserts. Genome Res. 15, 1061–1072 (doi:10.1101/gr.4004805) 16024824 10.1101/gr.4004805 PMC1182218

[14] Guo G Bauer S Hecht J Schulz MH Busche A Robinson PN 2008 A short ultraconserved sequence drives transcription from an alternate FBN1 promoter. Int. J. Biochem. Cell Biol. 40, 638–650 (doi:10.1016/j.biocel.2007.09.004) 17996480 10.1016/j.biocel.2007.09.004

[15] Sakuraba Y 2008 Identification and characterization of new long conserved noncoding sequences in vertebrates. Mamm. Genome 19, 703–712 (doi:10.1007/s00335-008-9152-7) 19015917 10.1007/s00335-008-9152-7

[16] Dermitzakis ET Kirkness E Schwarz S Birney E Reymond A Antonarakis SE 2004 Comparison of human chromosome 21 conserved nongenic sequences (CNGs) with the mouse and dog genomes shows that their selective constraint is independent of their genic environment. Genome Res. 14, 852–859 (doi:10.1101/gr.1934904) 15078857 10.1101/gr.1934904 PMC479112

[17] Engström PG Fredman D Lenhard B 2008 Ancora: a web resource for exploring highly conserved noncoding elements and their association with developmental regulatory genes. Genome Biol. 9, R34 (doi:10.1186/gb-2008-9-2-r34) 18279518 10.1186/gb-2008-9-2-r34 PMC2374709

[18] Persampieri J Ritter DI Lees D Lehoczky J Li Q Guo S Chuang JH 2008 cneViewer: a database of conserved non-coding elements for studies of tissue-specific gene regulation. Bioinformatics 24, 2418–2419 (doi:10.1093/bioinformatics/btn443) 18718943 10.1093/bioinformatics/btn443 PMC2562007

[19] Woolfe A Goode DK Cooke J Callaway H Smith S Snell P McEwen GK Elgar G 2007 CONDOR: a database resource of developmentally associated conserved non-coding elements. BMC Dev. Biol. 7, 100 (doi:10.1186/1471-213X-7-100) 17760977 10.1186/1471-213X-7-100 PMC2020477

[20] Dimitrieva S Bucher P 2013 UCNEbase—a database of ultraconserved non-coding elements and genomic regulatory blocks. Nucleic Acids Res. 41(Database issue), D101–D109 (doi:10.1093/nar/gks1092) 23193254 10.1093/nar/gks1092 PMC3531063

[21] Visel A Minovitsky S Dubchak I Pennacchio LA 2007 VISTA Enhancer Browser—a database of tissue-specific human enhancers. Nucleic Acids Res. 35(Suppl. 1), D88–D92 (doi:10.1093/nar/gkl822) 17130149 10.1093/nar/gkl822 PMC1716724

[22] Nobrega MA Ovcharenko I Afzal V Rubin EM 2003 Scanning human gene deserts for long-range enhancers. Science 302, 413 (doi:10.1126/science.1088328) 14563999 10.1126/science.1088328

[23] Visel A 2008 Ultraconservation identifies a small subset of extremely constrained developmental enhancers. Nat. Genet. 40, 158–160 (doi:10.1038/ng.2007.55) 18176564 10.1038/ng.2007.55 PMC2647775

[24] Clarke SL VanderMeer JE Wenger AM Schaar BT Ahituv N Bejerano G 2012 Human developmental enhancers conserved between deuterostomes and protostomes. PLoS Genet. 8, e1002852 (doi:10.1371/journal.pgen.1002852) 22876195 10.1371/journal.pgen.1002852 PMC3410860

[25] Kritsas K Wuest SE Hupalo D Kern AD Wicker T Grossniklaus U 2012 Computational analysis and characterization of UCE-like elements (ULEs) in plant genomes. Genome Res. 22, 2455–2466 (doi:10.1101/gr.129346.111) 22987666 10.1101/gr.129346.111 PMC3514675

[26] Plessy C Dickmeis T Chalmel F Strähle U 2005 Enhancer sequence conservation between vertebrates is favoured in developmental regulator genes. Trends Genet. 21, 207–210 (doi:10.1016/j.tig.2005.02.006) 15797614 10.1016/j.tig.2005.02.006

[27] Vavouri T Walter K Gilks WR Lehner B Elgar G 2007 Parallel evolution of conserved non-coding elements that target a common set of developmental regulatory genes from worms to humans. Genome Biol. 8, R15 (doi:10.1186/gb-2007-8-2-r15) 17274809 10.1186/gb-2007-8-2-r15 PMC1852409

[28] Goode DK Snell P Smith SF Cooke JE Elgar G 2005 Highly conserved regulatory elements around the SHH gene may contribute to the maintenance of conserved synteny across human chromosome 7q36.3. Genomics 86, 172–181 (doi:10.1016/j.ygeno.2005.04.006) 15939571 10.1016/j.ygeno.2005.04.006

[29] Irimia M 2012 Extensive conservation of ancient microsynteny across metazoans due to *cis*-regulatory constraints. Genome Res. 22, 2356–2367 (doi:10.1101/gr.139725.112) 22722344 10.1101/gr.139725.112 PMC3514665

[30] Kikuta H 2007 Genomic regulatory blocks encompass multiple neighboring genes and maintain conserved synteny in vertebrates. Genome Res. 17, 545–555 (doi:10.1101/gr.6086307) 17387144 10.1101/gr.6086307 PMC1855176

[31] Engström PG Ho Sui SJ Drivenes O Becker TS Lenhard B 2007 Genomic regulatory blocks underlie extensive microsynteny conservation in insects. Genome Res. 17, 1898–1908 (doi:10.1101/gr.6669607) 17989259 10.1101/gr.6669607 PMC2099597

[32] Maeso I 2012 An ancient genomic regulatory block conserved across bilaterians and its dismantling in tetrapods by retrogene replacement. Genome Res. 22, 642–655 (doi:10.1101/gr.132233.111) 22234889 10.1101/gr.132233.111 PMC3317147

[33] Lenhard B Sandelin A Carninci P 2012 Metazoan promoters: emerging characteristics and insights into transcriptional regulation. Nat. Rev. Genet. 13, 233–245 (doi:10.1038/nrg3163) 22392219 10.1038/nrg3163

[34] Marinić M Aktas T Ruf S Spitz F 2013 An integrated holo-enhancer unit defines tissue and gene specificity of the Fgf8 regulatory landscape. Dev. Cell. 11, 530–542 (doi:10.1016/j.devcel.2013.01.025) 23453598 10.1016/j.devcel.2013.01.025

[35] Lettice LA 2003 A long-range Shh enhancer regulates expression in the developing limb and fin and is associated with preaxial polydactyly. Hum. Mol. Genet. 12, 1725–1735 (doi:10.1093/hmg/ddg180) 12837695 10.1093/hmg/ddg180

[36] Sheng Y Previti C 2011 Genomic features and computational identification of human microRNAs under long-range developmental regulation. BMC Genomics 12, 270 (doi:10.1186/1471-2164-12-270) 21619633 10.1186/1471-2164-12-270 PMC3123655

[37] Derti A Roth FP Church GM Wu C-T 2006 Mammalian ultraconserved elements are strongly depleted among segmental duplications and copy number variants. Nat. Genet. 38, 1216–1220 (doi:10.1038/ng1888) 16998490 10.1038/ng1888

[38] Simons C Pheasant M Makunin IV Mattick JS 2006 Transposon-free regions in mammalian genomes. Genome Res. 16, 164–172 (doi:10.1101/gr.4624306) 16365385 10.1101/gr.4624306 PMC1361711

[39] McEwen GK Goode DK Parker HJ Woolfe A Callaway H Elgar G 2009 Early evolution of conserved regulatory sequences associated with development in vertebrates. PLoS Genet. 5, e1000762 (doi:10.1371/journal.pgen.1000762) 20011110 10.1371/journal.pgen.1000762 PMC2781166

[40] Hufton AL Mathia S Braun H Georgi U Lehrach H Vingron M Poustka AJ Panopoulou G 2009 Deeply conserved chordate noncoding sequences preserve genome synteny but do not drive gene duplicate retention. Genome Res. 19, 2036–2051 (doi:10.1101/gr.093237.109) 19704032 10.1101/gr.093237.109 PMC2775584

[41] Manzanares M Wada H Itasaki N Trainor PA Krumlauf R Holland PW 2000 Conservation and elaboration of Hox gene regulation during evolution of the vertebrate head. Nature 408, 854–857 (doi:10.1038/35048570) 11130723 10.1038/35048570

[42] Natale A Sims C Chiusano ML Amoroso A D'Aniello E Fucci L Krumlauf R Branno M Locascio A 2011 Evolution of anterior Hox regulatory elements among chordates. BMC Evol. Biol. 11, 330 (doi:10.1186/1471-2148-11-330) 22085760 10.1186/1471-2148-11-330 PMC3227721

[43] Ryu T Seridi L Ravasi T 2012 The evolution of ultraconserved elements with different phylogenetic origins. BMC Evol. Biol. 12, 236 (doi:10.1186/1471-2148-12-236) 23217155 10.1186/1471-2148-12-236 PMC3556307

[44] Glazov EA Pheasant M McGraw EA Bejerano G Mattick JS 2005 Ultraconserved elements in insect genomes: a highly conserved intronic sequence implicated in the control of homothorax mRNA splicing. Genome Res. 15, 800–808 (doi:10.1101/gr.3545105) 15899965 10.1101/gr.3545105 PMC1142470

[45] Baxter L 2012 Conserved noncoding sequences highlight shared components of regulatory networks in dicotyledonous plants. Plant Cell. 24, 3949–3965 (doi:10.1105/tpc.112.103010) 23110901 10.1105/tpc.112.103010 PMC3517229

[46] Walter K Abnizova I Elgar G Gilks WR 2005 Striking nucleotide frequency pattern at the borders of highly conserved vertebrate non-coding sequences. Trends Genet. 21, 436–440 (doi:10.1016/j.tig.2005.06.003) 15979195 10.1016/j.tig.2005.06.003

[47] Hare EE Peterson BK Iyer VN Meier R Eisen MB 2008 Sepsid even-skipped enhancers are functionally conserved in *Drosophila* despite lack of sequence conservation. PLoS Genet. 4, e1000106 (doi:10.1371/journal.pgen.1000106) 18584029 10.1371/journal.pgen.1000106 PMC2430619

[48] Kulkarni MM Arnosti DN 2003 Information display by transcriptional enhancers. Development 130, 6569–6575 (doi:10.1242/dev.00890) 14660545 10.1242/dev.00890

[49] Müller F Williams DW Kobolák J Gauvry L Goldspink G Orbán L Maclean N 1997 Activator effect of coinjected enhancers on the muscle-specific expression of promoters in zebrafish embryos. Mol. Reprod. Dev. 47, 404–412 (doi:10.1002/(SICI)1098-2795(199708)47:4<404::AID-MRD6>3.0.CO;2-O) 9211424 10.1002/(SICI)1098-2795(199708)47:4<404::AID-MRD6>3.0.CO;2-O

[50] Prabhakar S Poulin F Shoukry M Afzal V Rubin EM Couronne O Pennacchio LA 2006 Close sequence comparisons are sufficient to identify human *cis*-regulatory elements. Genome Res. 16, 855–863 (doi:10.1101/gr.4717506) 16769978 10.1101/gr.4717506 PMC1484452

[51] Royo JL 2011 Dissecting the transcriptional regulatory properties of human chromosome 16 highly conserved non-coding regions. PLoS ONE 6, e24824 (doi:10.1371/journal.pone.0024824) 21935474 10.1371/journal.pone.0024824 PMC3172297

[52] De Grassi A Segala C Iannelli F Volorio S Bertario L Radice P Bernard L Ciccarelli FD Hastie N 2010 Ultradeep sequencing of a human ultraconserved region reveals somatic and constitutional genomic instability. PLoS Biol. 8, e1000275 (doi:10.1371/journal.pbio.1000275) 20052272 10.1371/journal.pbio.1000275 PMC2794366

[53] Daniel C Venø MT Ekdahl Y Kjems J Ohman M 2012 A distant *cis* acting intronic element induces site-selective RNA editing. Nucleic Acids Res. 40, 9876–9886 (doi:10.1093/nar/gks691) 22848101 10.1093/nar/gks691 PMC3479170

[54] Ritter DI Li Q Kostka D Pollard KS Guo S Chuang JH 2010 The importance of being *cis*: evolution of orthologous fish and mammalian enhancer activity. Mol. Biol. Evol. 27, 2322–2332 (doi:10.1093/molbev/msq128) 20494938 10.1093/molbev/msq128 PMC3107594

[55] Royo JL 2011 Transphyletic conservation of developmental regulatory state in animal evolution. Proc. Natl Acad. Sci. USA 108, 14 186–14 191 (doi:10.1073/pnas.1109037108) 10.1073/pnas.1109037108 PMC3161536 21844364

[56] Nelson AC Wardle FC 2013 Conserved non-coding elements and *cis* regulation: actions speak louder than words. Development 140, 1385–1395 (doi:10.1242/dev.084459) 23482485 10.1242/dev.084459

[57] Vavouri T Lehner B 2009 Conserved noncoding elements and the evolution of animal body plans. Bioessays 31, 727–735 (doi:10.1002/bies.200900014) 19492354 10.1002/bies.200900014

[58] Sanges R 2013 Highly conserved elements discovered in vertebrates are present in non-syntenic loci of tunicates, act as enhancers and can be transcribed during development. Nucleic Acids Res. 41, 3600–3618 (doi:10.1093/nar/gkt030) 23393190 10.1093/nar/gkt030 PMC3616699

[59] Ahituv N Zhu Y Visel A Holt A Afzal V Pennacchio LA Rubin EM 2007 Deletion of ultraconserved elements yields viable mice. PLoS Biol. 5, e234 (doi:10.1371/journal.pbio.0050234) 17803355 10.1371/journal.pbio.0050234 PMC1964772

[60] Hong J-W Hendrix DA Levine MS 2008 Shadow enhancers as a source of evolutionary novelty. Science 321, 1314 (doi:10.1126/science.1160631) 18772429 10.1126/science.1160631 PMC4257485

[61] Frankel N Davis GK Vargas D Wang S Payre F Stern DL 2010 Phenotypic robustness conferred by apparently redundant transcriptional enhancers. Nature 466, 490–493 (doi:10.1038/nature09158) 20512118 10.1038/nature09158 PMC2909378

[62] Franchini LF López-Leal R Nasif S Beati P Gelman DM Low MJ de Souza FJS Rubinstein M 2011 Convergent evolution of two mammalian neuronal enhancers by sequential exaptation of unrelated retroposons. Proc. Natl Acad. Sci. USA 108, 15 270–15 275 (doi:10.1073/pnas.1104997108) 10.1073/pnas.1104997108 PMC3174587 21876128

[63] Fisher S Grice EA Vinton RM Bessling SL McCallion AS 2006 Conservation of RET regulatory function from human to zebrafish without sequence similarity. Science 312, 276–279 (doi:10.1126/science.1124070) 16556802 10.1126/science.1124070

[64] Alexander RP Fang G Rozowsky J Snyder M Gerstein MB 2010 Annotating non-coding regions of the genome. Nat. Rev. Genet. 11, 559–571 (doi:10.1038/nrg2814) 20628352 10.1038/nrg2814

[65] Schmidt D 2010 Five-vertebrate ChIP-seq reveals the evolutionary dynamics of transcription factor binding. Science 328, 1036–1040 (doi:10.1126/science.1186176) 20378774 10.1126/science.1186176 PMC3008766

[66] Visel A Rubin EM Pennacchio LA 2009 Genomic views of distant-acting enhancers. Nature 461, 199–205 (doi:10.1038/nature08451) 19741700 10.1038/nature08451 PMC2923221

[67] Maurano MT 2012 Systematic localization of common disease-associated variation in regulatory DNA. Science 337, 1190–1195 (doi:10.1126/science.1222794) 22955828 10.1126/science.1222794 PMC3771521

[68] Schaub MA Boyle AP Kundaje A Batzoglou S Snyder M 2012 Linking disease associations with regulatory information in the human genome. Genome Res. 22, 1748–1759 (doi:10.1101/gr.136127.111) 22955986 10.1101/gr.136127.111 PMC3431491

[69] Ragvin A 2010 Long-range gene regulation links genomic type 2 diabetes and obesity risk regions to HHEX, SOX4, and IRX3. Proc. Natl Acad. Sci. USA 107, 775–780 (doi:10.1073/pnas.0911591107) 20080751 10.1073/pnas.0911591107 PMC2818943

[70] Drake JA 2006 Conserved noncoding sequences are selectively constrained and not mutation cold spots. Nat. Genet. 38, 223–227 (doi:10.1038/ng1710) 16380714 10.1038/ng1710

[71] Katzman S Kern AD Bejerano G Fewell G Fulton L Wilson RK Salama SR Haussler D 2007 Human genome ultraconserved elements are ultraselected. Science 317, 915 (doi:10.1126/science.1142430) 17702936 10.1126/science.1142430

[72] Casillas S Barbadilla A Bergman CM 2007 Purifying selection maintains highly conserved noncoding sequences in *Drosophila*. Mol. Biol. Evol. 24, 2222–2234 (doi:10.1093/molbev/msm150) 17646256 10.1093/molbev/msm150

[73] McEwen GK Woolfe A Goode D Vavouri T Callaway H Elgar G 2006 Ancient duplicated conserved noncoding elements in vertebrates: a genomic and functional analysis. Genome Res. 16, 451–665 (doi:10.1101/gr.4143406) 16533910 10.1101/gr.4143406 PMC1457030

[74] Dong X Fredman D Lenhard B 2009 Synorth: exploring the evolution of synteny and long-range regulatory interactions in vertebrate genomes. Genome Biol. 10, R86 (doi:10.1186/gb-2009-10-8-r86) 19698106 10.1186/gb-2009-10-8-r86 PMC2745767

[75] Dong X Navratilova P Fredman D Drivenes O Becker TS Lenhard B 2010 Exonic remnants of whole-genome duplication reveal *cis*-regulatory function of coding exons. Nucleic Acids Res. 38, 1071–1085 (doi:10.1093/nar/gkp1124) 19969543 10.1093/nar/gkp1124 PMC2831330

[76] Lampe X Samad OA Guiguen A Matis C Remacle S Picard JJ Rijli FM Rezsohazy R 2008 An ultraconserved Hox-Pbx responsive element resides in the coding sequence of Hoxa2 and is active in rhombomere 4. Nucleic Acids Res. 36, 3214–3225 (doi:10.1093/nar/gkn148) 18417536 10.1093/nar/gkn148 PMC2425489

[77] Lowe CB Bejerano G Haussler D 2007 Thousands of human mobile element fragments undergo strong purifying selection near developmental genes. Proc. Natl Acad. Sci. USA 104, 8005–8010 (doi:10.1073/pnas.0611223104) 17463089 10.1073/pnas.0611223104 PMC1876562

[78] Kamal M Xie X Lander ES 2006 A large family of ancient repeat elements in the human genome is under strong selection. Proc. Natl Acad. Sci. USA 103, 2740–2745 (doi:10.1073/pnas.0511238103) 16477033 10.1073/pnas.0511238103 PMC1413850

[79] de Souza FSJ Franchini LF Rubinstein M 2013 Exaptation of transposable elements into novel *cis*-regulatory elements: is the evidence always strong? Mol. Biol. Evol. 30, 1239–1251 (doi:10.1093/molbev/mst045) 23486611 10.1093/molbev/mst045 PMC3649676

[80] Bourque G 2008 Evolution of the mammalian transcription factor binding repertoire via transposable elements. Genome Res. 18, 1752–1762 (doi:10.1101/gr.080663.108) 18682548 10.1101/gr.080663.108 PMC2577865

[81] Lowe CB Haussler D 2012 29 mammalian genomes reveal novel exaptations of mobile elements for likely regulatory functions in the human genome. PLoS ONE 7, e43128 (doi:10.1371/journal.pone.0043128) 22952639 10.1371/journal.pone.0043128 PMC3428314

[82] Wang J Lee AP Kodzius R Brenner S Venkatesh B 2009 Large number of ultraconserved elements were already present in the jawed vertebrate ancestor. Mol. Biol. Evol. 26, 487–490 (doi:10.1093/molbev/msn278) 19052148 10.1093/molbev/msn278

[83] Stephen S Pheasant M Makunin IV Mattick JS 2008 Large-scale appearance of ultraconserved elements in tetrapod genomes and slowdown of the molecular clock. Mol. Biol. Evol. 25, 402–408 (doi:10.1093/molbev/msm268) 18056681 10.1093/molbev/msm268

[84] Kim SY Pritchard JK 2007 Adaptive evolution of conserved noncoding elements in mammals. PLoS Genet. 3, 1572–1586 (doi:10.1371/journal.pgen.0030147) 17845075 10.1371/journal.pgen.0030147 PMC1971121

[85] Lowe CB 2011 Three periods of regulatory innovation during vertebrate evolution. Science 333, 1019–1024 (doi:10.1126/science.1202702) 21852499 10.1126/science.1202702 PMC3511857

[86] Takahashi M Saitou N 2012 Identification and characterization of lineage-specific highly conserved noncoding sequences in mammalian genomes. Genome Biol. Evol. 4, 641–657 (doi:10.1093/gbe/evs035) 22505575 10.1093/gbe/evs035 PMC3381673

[87] McCormack JE Faircloth BC Crawford NG Gowaty PA Brumfield RT Glenn TC 2012 Ultraconserved elements are novel phylogenomic markers that resolve placental mammal phylogeny when combined with species-tree analysis. Genome Res. 22, 746–754 (doi:10.1101/gr.125864.111) 22207614 10.1101/gr.125864.111 PMC3317156

[88] Faircloth BC McCormack JE Crawford NG Harvey MG Brumfield RT Glenn TC 2012 Ultraconserved elements anchor thousands of genetic markers spanning multiple evolutionary timescales. Syst. Biol. 61, 717–726 (doi:10.1093/sysbio/sys004) 22232343 10.1093/sysbio/sys004

[89] Ovcharenko I 2008 Widespread ultraconservation divergence in primates. Mol. Biol. Evol. 25, 1668–1676 (doi:10.1093/molbev/msn116) 18492662 10.1093/molbev/msn116 PMC2464743

[90] McLean CY 2011 Human-specific loss of regulatory DNA and the evolution of human-specific traits. Nature 471, 216–219 (doi:10.1038/nature09774) 21390129 10.1038/nature09774 PMC3071156

[91] Chan YF 2010 Adaptive evolution of pelvic reduction in sticklebacks by recurrent deletion of a Pitx1 enhancer. Science 327, 302–305 (doi:10.1126/science.1182213) 20007865 10.1126/science.1182213 PMC3109066

[92] McLean C Bejerano G 2008 Dispensability of mammalian DNA. Genome Res. 18, 1743–1751 (doi:10.1101/gr.080184.108) 18832441 10.1101/gr.080184.108 PMC2577864

[93] Hiller M Schaar BT Bejerano G 2012 Hundreds of conserved non-coding genomic regions are independently lost in mammals. Nucleic Acids Res. 40, 11 463–11 476 (doi:10.1093/nar/gks905) 23042682 10.1093/nar/gks905 PMC3526296

[94] Janes DE 2011 Reptiles and mammals have differentially retained long conserved noncoding sequences from the amniote ancestor. Genome Biol. Evol. 3, 102–113 (doi:10.1093/gbe/evq087) 21183607 10.1093/gbe/evq087 PMC3035132

[95] Kalay G Wittkopp PJ 2010 Nomadic enhancers: tissue-specific *cis*-regulatory elements of *yellow* have divergent genomic positions among *Drosophila* species. PLoS Genet. 6, e1001222 (doi:10.1371/journal.pgen.1001222) 21151964 10.1371/journal.pgen.1001222 PMC2996884

[96] Eichenlaub MP Ettwiller L 2011 De novo genesis of enhancers in vertebrates. PLoS Biol. 9, e1001188 (doi:10.1371/journal.pbio.1001188) 22069375 10.1371/journal.pbio.1001188 PMC3206014

[97] Smith NGC Brandström M Ellegren H 2004 Evidence for turnover of functional noncoding DNA in mammalian genome evolution. Genomics 84, 806–813 (doi:10.1016/j.ygeno.2004.07.012) 15475259 10.1016/j.ygeno.2004.07.012

[98] Chatterjee S Bourque G Lufkin T 2011 Conserved and non-conserved enhancers direct tissue specific transcription in ancient germ layer specific developmental control genes. BMC Dev. Biol. 11, 63 (doi:10.1186/1471-213X-11-63) 22011226 10.1186/1471-213X-11-63 PMC3210094

[99] Spitz F Furlong EEM 2012 Transcription factors: from enhancer binding to developmental control. Nat. Rev. Genet. 13, 613–626 (doi:10.1038/nrg3207) 22868264 10.1038/nrg3207

[100] Panne D 2008 The enhanceosome. Curr. Opin. Struct. Biol. 18, 236–242 (doi:10.1016/j.sbi.2007.12.002) 18206362 10.1016/j.sbi.2007.12.002

[101] Kwasnieski JC Mogno I Myers CA Corbo JC Cohen BA 2012 Complex effects of nucleotide variants in a mammalian *cis*-regulatory element. Proc. Natl Acad. Sci. USA 109, 19 498–19 503 (doi:10.1073/pnas.1210678109) 10.1073/pnas.1210678109 PMC3511131 23129659

[102] Frankel N Erezyilmaz DF McGregor AP Wang S Payre F Stern DL 2011 Morphological evolution caused by many subtle-effect substitutions in regulatory DNA. Nature 474, 598–603 (doi:10.1038/nature10200) 21720363 10.1038/nature10200 PMC3170772

[103] ENCODE Project Consortium, Dunham I 2012 An integrated encyclopedia of DNA elements in the human genome. Nature 489, 57–74 (doi:10.1038/nature11247) 22955616 10.1038/nature11247 PMC3439153

[104] Neph S 2012 An expansive human regulatory lexicon encoded in transcription factor footprints. Nature 489, 83–90 (doi:10.1038/nature11212) 22955618 10.1038/nature11212 PMC3736582

[105] Visel A 2009 ChIP-seq accurately predicts tissue-specific activity of enhancers. Nature 457, 854–858 (doi:10.1038/nature07730) 19212405 10.1038/nature07730 PMC2745234

[106] Komisarczuk AZ Kawakami K Becker TS 2009 *Cis*-regulation and chromosomal rearrangement of the fgf8 locus after the teleost/tetrapod split. Dev. Biol. 336, 301–312 (doi:10.1016/j.ydbio.2009.09.029) 19782672 10.1016/j.ydbio.2009.09.029

[107] Davidson EH 2011 Evolutionary bioscience as regulatory systems biology. Dev. Biol. 357, 35–40 (doi:10.1016/j.ydbio.2011.02.004) 21320483 10.1016/j.ydbio.2011.02.004 PMC3135751

[108] Kalinka AT 2010 Gene expression divergence recapitulates the developmental hourglass model. Nature 468, 811–814 (doi:10.1038/nature09634) 21150996 10.1038/nature09634

[109] Duboule D 1994 Temporal colinearity and the phylotypic progression: a basis for the stability of a vertebrate Bauplan and the evolution of morphologies through heterochrony. Dev. Suppl. 1994, 135–142 7579514

[110] Bogdanovic O 2012 Dynamics of enhancer chromatin signatures mark the transition from pluripotency to cell specification during embryogenesis. Genome Res. 22, 2043–2053 (doi:10.1101/gr.134833.111) 22593555 10.1101/gr.134833.111 PMC3460198

[111] Chiang CWK Derti A Schwartz D Chou MF Hirschhorn JN Wu C-T 2008 Ultraconserved elements: analyses of dosage sensitivity, motifs and boundaries. Genetics 180, 2277–2293 (doi:10.1534/genetics.108.096537) 18957701 10.1534/genetics.108.096537 PMC2600958

[112] Robyr D 2011 Chromosome conformation capture uncovers potential genome-wide interactions between human conserved non-coding sequences. PLoS ONE 6, e17634 (doi:10.1371/journal.pone.0017634) 21408183 10.1371/journal.pone.0017634 PMC3049788

[113] Chambers EV Bickmore WA Semple CA 2013 Divergence of mammalian higher order chromatin structure is associated with developmental loci. PLoS Comput. Biol. 9, e1003017 (doi:10.1371/journal.pcbi.1003017) 23592965 10.1371/journal.pcbi.1003017 PMC3617018

[114] Hammoud SS Nix DA Zhang H Purwar J Carrell DT Cairns BR 2009 Distinctive chromatin in human sperm packages genes for embryo development. Nature 460, 473–478 (doi:10.1038/nature08162) 19525931 10.1038/nature08162 PMC2858064

[115] Vavouri T Lehner B 2011 Chromatin organization in sperm may be the major functional consequence of base composition variation in the human genome. PLoS Genet. 7, e1002036 (doi:10.1371/journal.pgen.1002036) 21490963 10.1371/journal.pgen.1002036 PMC3072381

[116] Wu S-F Zhang H Cairns BR 2011 Genes for embryo development are packaged in blocks of multivalent chromatin in zebrafish sperm. Genome Res. 21, 578–589 (doi:10.1101/gr.113167.110) 21383318 10.1101/gr.113167.110 PMC3065705

[117] Ng J-H 2013 *In vivo* epigenomic profiling of germ cells reveals germ cell molecular signatures. Dev. Cell 24, 324–333 (doi:10.1016/j.devcel.2012.12.011) 23352811 10.1016/j.devcel.2012.12.011

